# Neutralization of cholera toxin with nanoparticle decoys for treatment of cholera

**DOI:** 10.1371/journal.pntd.0006266

**Published:** 2018-02-22

**Authors:** Soumita Das, Pavimol Angsantikul, Christine Le, Denny Bao, Yukiko Miyamoto, Weiwei Gao, Liangfang Zhang, Lars Eckmann

**Affiliations:** 1 Department of Pathology, University of California, San Diego, La Jolla, California, United States of America; 2 Department of Nanoengineering, University of California, San Diego, La Jolla, California, United States of America; 3 Department of Medicine, University of California, San Diego, La Jolla, California, United States of America; College of Health Sciences, Bayero University Kano, NIGERIA

## Abstract

Diarrheal diseases are a major cause of morbidity and mortality worldwide. In many cases, antibiotic therapy is either ineffective or not recommended due to concerns about emergence of resistance. The pathogenesis of several of the most prevalent infections, including cholera and enteroxigenic *Escherichia coli*, is dominated by enterotoxins produced by lumen-dwelling pathogens before clearance by intestinal defenses. Toxins gain access to the host through critical host receptors, making these receptors attractive targets for alternative antimicrobial strategies that do not rely on conventional antibiotics. Here, we developed a new nanotechnology strategy as a countermeasure against cholera, one of the most important and prevalent toxin-mediated enteric infections. The key host receptor for cholera toxin, monosialotetrahexosylganglioside (GM1), was coated onto the surface of polymeric nanoparticles. The resulting GM1-polymer hybrid nanoparticles were shown to function as toxin decoys by selectively and stably binding cholera toxin, and neutralizing its actions on epithelial cells *in vitro* and *in vivo*. Furthermore, the GM1-coated nanoparticle decoys attenuated epithelial 3’,5’-cyclic adenosine monophosphate production and fluid responses to infection with live *Vibrio cholera* in cell culture and a murine infection model. Together, these studies illustrate that the new nanotechnology-based platform can be employed as a non-traditional antimicrobial strategy for the management of enteric infections with enterotoxin-producing pathogens.

## Author summary

Diarrheal diseases are a major cause of suffering and death in the world, particularly in tropical regions with limited health care resources. Many of the most important diarrhea-causing microbes produce toxins that activate fluid secretion in the gut. A prototype pathogen in this category is the cause of cholera, *Vibrio cholerae*, which is characterized by profuse diarrhea and severe electrolyte disturbances due to the release of cholera toxin. Although treatment with fluids by mouth or injection can save patients from death, they still experience the devastating symptoms of the disease. In the present study, we have developed a new intervention strategy with engineered nanoparticles, particulates than are smaller than one millionth of a meter, which can neutralize cholera toxin in the gut before it can cause the characteristic disease manifestations. This strategy represents a novel interventional approach whose mechanism of action is different from currently existing therapies, thus significantly broadening the medical armamentarium against cholera and perhaps other gut infections that cause diseases dominated by toxin production.

## Introduction

Diarrheal diseases are a major cause of morbidity and mortality in developing regions, with an estimated 3–5 million cases and over 100,000 deaths per year [1,2]. Diarrhea accounts for 1 in 9 childhood deaths worldwide, making it the second leading cause of death among children under the age of five. As an example, in diarrheal patients attending a hospital in Mirpur, Bangladesh, *Vibrio cholerae* was found to be the causative agent of diarrheal disease in 23% of patients [3]. Current treatments involve rehydration with oral or intravenous replacement electrolyte solutions [4,5]. While this method has reduced mortality rates in children with acute diarrheal diseases, in general, stool volume and diarrheal durations are not decreased [6]. Administration of antibiotics in conjunction with electrolyte solutions can reduce the volume and duration of diarrhea [7], but extensive use of antibiotics may lead to the emergence of antibiotic-resistant strains of bacteria, threatening the utility of existing antibiotics [8]. Therefore, an urgent need exists to develop alternative treatments.

*V*. *cholerae* is usually contracted through ingestion of contaminated water or food in which the bacterium is present [5]. While bacterial colonization is limited to the lumen and epithelial surface of the intestinal tract, the disease symptoms are primarily caused by bacterially produced toxins. Most prominently, *V*. *cholerae* secretes cholera toxin (CT), which is composed of an A subunit responsible for toxicity and a pentameric B subunit (CTB) responsible for receptor binding [9]. CTB binds to GM1 gangliosides on the surface of intestinal epithelial cells, which subsequently leads to endocytosis of the entire protein complex [10]. The A1 subunit (CTA1) is cleaved from the rest of the toxin through the reduction of a disulfide bond [11]. CTA1 then catalyzes ADP-ribosylation of the G_Sα_ protein [12], leading to its activation, stimulation of adenylyl cyclases, and a sustained increase in epithelial cyclic AMP levels [13]. This series of events culminates in a massive efflux of chloride ions and an inhibition of sodium absorption by the epithelium, which leads to the rapid outflow of water into the intestinal lumen, and the attending severe diarrhea and dehydration [14].

Since the GM1 ganglioside host receptors play a key role in the CT-mediated pathogenesis of cholera, it constitutes an attractive target for novel antimicrobial strategies [15]. The recent emergence of nanotechnology is beginning to have a profound impact on modern medicine [16]. Nanoparticle systems have shown to be superior in facilitating drug solubility, systemic circulation, and drug release, and in their ability for differential cell targeting compared to free drugs [17,18]. In addition, nanoparticles can be engineered to serve as decoys or sinks for microbial toxins, opening up new possibilities for treating toxin-mediated diseases [19,20]. To determine whether this concept can be applied to cholera as a prototypic model for intestinal diseases caused by enterotoxins, we set out to develop a nanotechnology-based strategy for CT neutralization and treatment of cholera. The work described here demonstrates that nanoparticle decoys are a promising new therapeutic avenue for toxin-mediated diarrheal diseases.

## Methods

### Ethics statement

Laboratory mice were used for parts of the study. Anesthesia was done with isoflurane inhalation, and buprenorphine was given before surgery for preventive pain management. Mice were euthanized by CO_2_ inhalation and cervical dislocation. All animal studies were reviewed and approved by the UC San Diego Institutional Animal Care and Use Committee.

### Preparation of nanoparticles

GM1 ganglioside-coated poly(lactic-co-glycolic acid) (PLGA) hybrid nanoparticles (GM1-NPs) were prepared by nanoprecipitation as previously described [18,21] with several modifications. Briefly, a PLGA stock solution was prepared by dissolving PLGA pellets (LACTEL Absorbable Polymers, Pelham, AL) in acetonitrile at a concentration of 2.5 mg/mL. A GM1 ganglioside stock solution was prepared by dissolving GM1 (Carbosynth, San Diego, CA) in deionized water at 10 mg/mL. To prepare the aqueous phase for GM1-NP synthesis, the desired amount of GM1 stock solution was added into deionized water to yield a final GM1 concentration of 10% (w/v) of the PLGA polymer. A predetermined volume of the PLGA solution was then added dropwise (1 ml/min) into the aqueous GM1 solution under gentle stirring. The nanoparticles were allowed to self-assemble for 2 h with continuous stirring while the organic solvent was allowed to evaporate under vacuum. To remove the remaining free molecules and organic solvent, the nanoparticle suspensions were washed in deionized water three times using an Amicon Ultra centrifuge filter (Millipore, Billerica, MA) with a molecular weight cut-off of 100 kDa. Nanoparticles were resuspended in deionized water and used immediately or stored at 4°C (up to 4 weeks) for later use.

As a control, PLGA nanoparticle cores (PLGA-NPs) were prepared with the nanoprecipitation method described above, but without GM1 in the aqueous solution. As another control, polyethylene glycol (PEG)-modified PLGA nanoparticles (PEG-NPs) were fabricated with a coat of 1,2-distearoyl-sn-glycero-3-phosphoethanolamine-N-[methoxy(polyethylene glycol)-2000] (DSPE-mPEG2000; average molecular weight 2.8 kDa, Laysan Bio, Inc., AL) through nanoprecipitation as previously described [21]. The aqueous phase contained a DSPE-mPEG2000 concentration of 10% (w/v) of the PLGA polymer. All stated concentrations for nanoparticles refer to the concentration of the PLGA polymer in the respective formulation.

### Physical characterization of nanoparticles

Nanoparticle stability was analyzed in deionized water and phosphate-buffered saline (PBS). For stability in water, nanoparticles were synthesized as described above at a final polymer concentration of 1 mg/mL. To test the stability in PBS, nanoparticles at 2 mg/mL in water were added to an equal volume of 2 × PBS. Particle size distribution and zeta-potential were measured by dynamic light scattering using a Malvern ZEN 3600 Zetasizer. Transmission electron microscopy of nanoparticles was done by depositing a suspension (2 mg/mL) on a glow-discharged, carbon-coated 400-mesh copper grid. The grid was washed with distilled water and stained with 1% (w/v) uranyl acetate. Imaging was carried out on a Zeiss Libra 120 PLUS energy filter transmission electron microscope.

### Cholera toxin binding studies

Binding of FITC-labeled CTB (Sigma-Aldrich, St. Louis, MO) was tested by incubating the different nanoparticle suspensions with 10 μg/mL CTB in in 400 μl of PBS (pH 7.2) for 30 min. Each sample was transferred to an Amicon Ultra centrifuge filter and centrifuged at 8,000 rpm in a Beckman Coulter microfuge 22R centrifuge for 5 min. Fluorescence was determined using a Synergy Mx fluorescent spectrophotometer (Biotek, Winooski, VT). Bound CTB was calculated with the formula: CTB bound (%) = (1- CTB in supernatant/total CTB input) × 100%. All experiments were performed in triplicate. Bound CTB was plotted against nanoparticle concentrations, and a curve was fitted with the binding-saturation equation in GraphPad Prism.

To investigate the CT binding and neutralization capability, 400 μl of PBS solution containing 1 or 0.25 mg/mL of nanoparticles was mixed with 5 μl of different concentrations of FITC-CTB, and incubated for 30 min at 37°C. Each sample was processed as described above, and bound CTB was calculated, plotted against CTB input concentrations, and fitted with a binding-saturation equation. To determine the binding capacity of different nanoparticle formulations, 400 μl of PBS solution containing 1 mg/mL of GM1-NPs or PEG-NPs were incubated with 10 μg/mL CTB for 30 min at 37°C. CTB incubated in PBS solution was used as the negative control. Each sample was processed and analyzed as described above, and the bound CTB was calculated.

To determine stability of toxin binding to the nanoparticles, 1 ml of a PBS solution containing 1 mg/mL of GM1-NPs was incubated with 10 μg/mL FITC-CTB for 30 min at 37°C. The sample were transferred to an Amicon Ultra centrifuge filter and centrifuged at 8,000 rpm for 5 min, and the CTB-loaded nanoparticles were resuspended in 1 ml of a pool of undiluted luminal content obtained from the small intestine of several male and female adult C57BL/6 mice. After 24 h incubation at 37°C, particles were dialyzed for 24 h against a PBS solution using a PTFE Dialyzer (Harvard Apparatus) and Nucleopore hydrophilic membrane (Whatman) with a molecular weight cut-off of 200 kDa. Retention of FITC-CTB on the nanoparticles was measured by fluorescence spectroscopy. GM1-NPs incubated in PBS and free CTB incubated in luminal content were used as positive and negative (background) control, respectively. Data are expressed as bound FITC-CTB after 24 h relative to the initial amount bound before incubation. Experiments were done in triplicate.

### Confocal microscopy of nanoparticles

To visualize nanoparticles and toxin colocalization, fluorescently-labeled GM1-NPs and PEG-NPs were prepared using 1,1’-dioctadecyl-3,3,3’,3’-tetramethylindo-dicarbocyanine perchlorate fluorescent dye (DiD; excitation/emission 644/665 nm; Life Technologies, Carlsbad, CA) for incorporation into the polymer solution at a concentration of 10 μg/mL during the preparation. Labeled GM1-NPs and PEG-NPs (1 mg/mL) were then incubated with 10 μg/mL CTB as described for the toxin binding studies. After 30 min incubation, nanoparticle solutions were washed three times in deionized water using an Amicon Ultra centrifuge filter, and the samples were visualized by confocal fluorescence microscopy using an Olympus FV1000 microscope with a 100x oil objective. To obtain stable images, the particles were dispersed in glycerol to significantly decrease their spontaneous movements.

### Cell culture studies

*Vibrio cholerae* strain N16961 (serogroup O1, biovar El Tor; ATCC) was grown overnight at 37°C in Luria Bertani broth supplemented with trimethylamine N-oxide without agitation at 37°C in an atmosphere of 5% CO_2_ and 95% air. These conditions have been shown to induce CT expression [22]. Human HCA7 colon cancer cells (ATCC) were grown in 75-cm^2^ culture flasks in Dulbecco’s modified Eagle’s medium (DMEM) supplemented with 10% fetal bovine serum and 1% penicillin-streptomycin at 37°C in 5% CO_2_ and 95% air. Cells were plated into 48-well plates at 5x10^5^ cells/well, and monolayers were grown overnight before experiments.

For toxin neutralization, we mixed different concentrations of CT (List Biological Laboratories, Campbell, CA) and nanoparticles, incubated for 1 h, and added the mixture to the cell monolayers. After 2 h, supernatants were collected and assayed for cAMP by enzyme immunoassay (Cyclic AMP ELISA Kit, Cayman Chemical Co., Ann Arbor, MI). All cAMP measurements were done without additional acetylation.

For neutralization experiments with live bacteria, nanoparticles were added to epithelial cell monolayers, which were then immediately inoculated with *V*. *cholerae* at a multiplicity-of-infection of 30, as determined by measuring optical density at 600 nm (OD_600_). After 2 h of infection, cAMP levels were determined in the supernatants by enzyme immunoassay.

### Intestinal ligated loops

Ligated loops of the mid-distal small intestine were prepared in anesthetized adult C57BL/6 mice as described previously [23]. Briefly, mice were fasted for 4–6 h before anesthesia and surgery, and given 0.1% buprenorphine for preventive pain management. After shaving and disinfection of the abdomen, a small abdominal incision was made, and a small intestinal loop was identified and ligated with two small surgical clips placed 2–3 cm apart. Agents were injected into the loop with a 30G needle in a 200 μl volume, and the abdominal cavity was closed with sutures. Loops were excised at different times, and the luminal loop volume was determined and related to the length of the loop. For CT tests, a solution of 12.5 μg/mL CT was incubated with and without 250 μg/mL GM1-NPs or PEG-NPs for 1 h at room temperature, and the mixture, or PBS as a control, was injected into the ligated loops. For tests with live bacteria, *V*. *cholerae* were prepared as described above, and injected in modified Luria Bertani broth at 10^5^ bacteria per loop, either alone or with 1.8 mg/loop of GM1-NPs or PEG-NPs. Broth alone was used as a control.

### Statistics

Data were analyzed using Prism 5 (GraphPad Software, La Jolla, USA). Means were compared with Student’s t-test or analysis of variance (Anova). P values <0.05 were considered as significant.

## Results

### Construction and physical characterization of GM1-coated nanoparticle decoys

**Fig 1:**
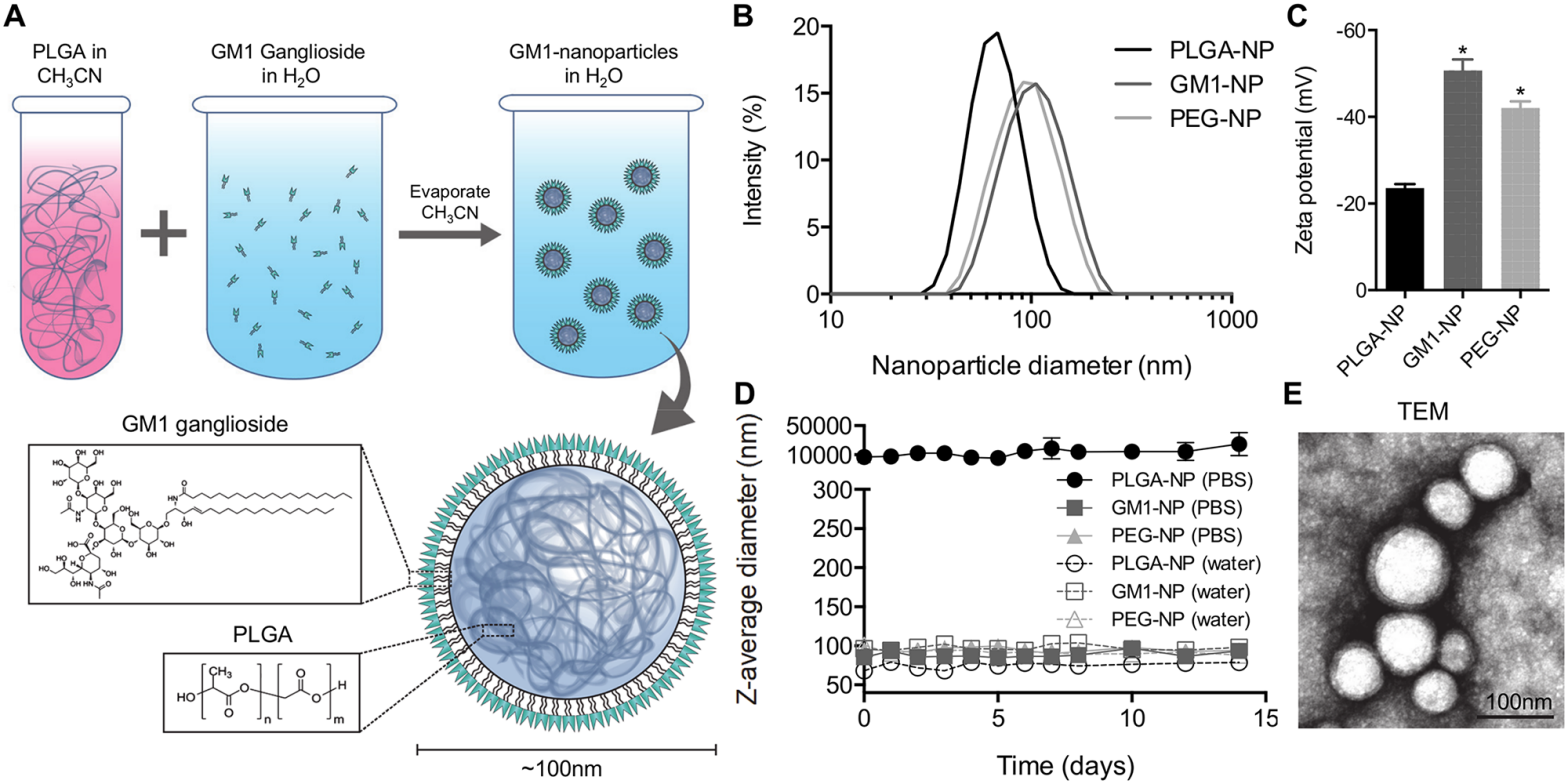
Preparation and physical characterization of GM1-coated nanoparticles. (A) Schematic of GM1-NP fabrication. Poly(lactic-co-glycolic acid) (PLGA) dissolved in acetonitrile (CH_3_CN) is added to an aqueous solution containing GM1. After acetonitrile evaporation, nanoparticles with a polymeric core and a lipid shell are formed. (B) Intensity-weighted size distribution of representative preparations of GM1-NPs and control PEG-NPs, and PLGA-NPs with a PLGA core but without a lipid shell. (C) Zeta potential of the indicated nanoparticle preparations (n = 3; mean ± SD; *p<0.05 vs. PLGA-NPs). (D) Nanoparticle size measurements over two weeks of incubation in distilled water or PBS (n = 3; mean ± SD). (E) Transmission electron micrograph of GM1-NPs.

Hybrid nanoparticles, comprised of a polymeric core and a lipid shell, combine the merits of both polymeric nanoparticles and liposomes while avoiding some of their limitations [18]. Compared to the aqueous cores of conventional liposomes, a solid polymeric core provides better control over the mechanical stability, particle morphology, size distribution, and drug release kinetics [21]. Therefore, we applied hybrid nanoparticle fabrication to the formulation of GM1-NPs as schematically outlined in Fig 1A. Briefly, an organic solution of PLGA as the polymeric core constituent was added dropwise under gentle stirring to a solution of GM1 in water to yield a final 1:2 volume ratio of organic to aqueous solution. The mixture was vortexed vigorously for 3 min followed by solvent evaporation under reduced pressure. The remaining organic solvent and free molecules were removed by centrifugation. As controls, two other types of nanoparticles were prepared: PLGA-NPs and PEG-NPs. PEG-NPs have a PLGA core coated with DSPE-mPEG2000 [24], a lipid modified with polyethylene glycol that is not expected to bind CT. PLGA-NPs are comprised of only a PLGA core without a lipid shell.

Analysis of the nanoparticles by dynamic light scattering revealed a narrow size distribution of the GM1-NPs, with a measured hydrodynamic diameter of ~100 nm (Fig 1B), which was similar to the previously reported size of the control PEG-NPs [21]. By comparison, the bare PLGA core had a slightly smaller diameter of ~75 nm (Fig 1B), suggesting that the increased size of the GM1-NPs was due to the additional GM1 gangliosides coated as an exterior layer onto the PLGA core. Furthermore, GM1-NPs as well as the control PEG-NPs, had a significantly more negative surface zeta-potential of -40 to -50 mV compared to -25 mV of PLGA-NPs (Fig 1C), which is also indicative of a differences in the nanoparticle surface characteristics due to the lipid coating of the GM1-NPs compared to the bare PLGA cores.

To test the stability of the nanoparticles in aqueous solution, they were incubated in water or PBS for up to two weeks and analyzed by dynamic light scattering. All three nanoparticle preparations were stable in water for the entire test period, but only the two lipid-coated preparations were stable in PBS, while the bare PLGA-NPs rapidly formed aggregates (Fig 1D). These findings clearly distinguished GM1-NPs from the uncoated PLGA-NPs and suggested that the GM1 layer surrounding the GM1-NPs can provide steric and electronic repulsion to prevent detrimental particle aggregation that would interfere with *in vitro* and *in vivo* studies. Transmission electron microscopy confirmed that the GM1-NPs were dispersed as single particles with a core/shell structure characteristic of a unilamellar membrane coating around a nanoparticle core (Fig 1E).

### Binding characteristics of cholera toxin to GM1 nanoparticle decoys

**Fig 2:**
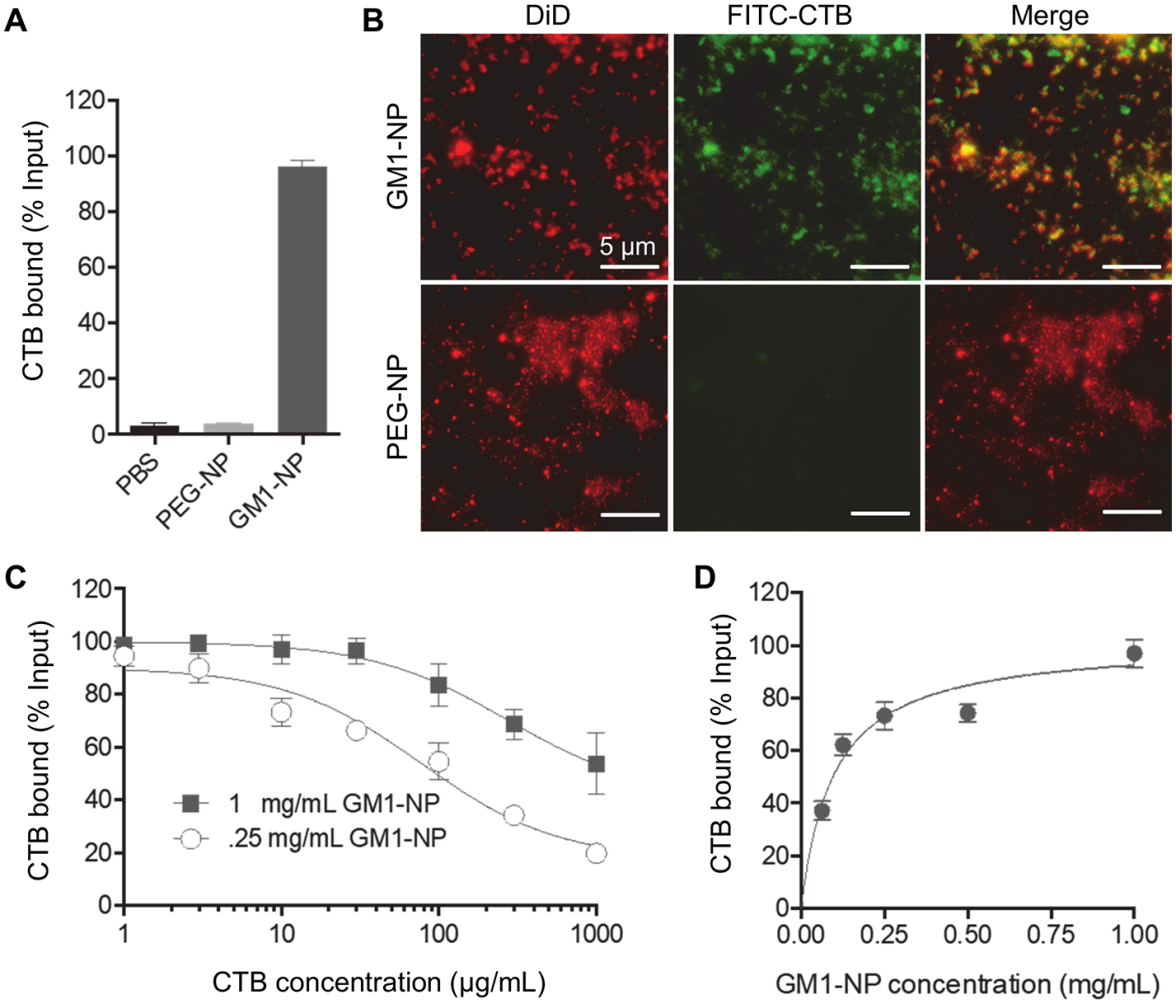
Specific binding of cholera toxin B subunit to GM1-NPs. (A) GM1-NPs and control PEG-NPs were incubated with FITC-labeled CTB for 30 min, washed by centrifugation, and analyzed by fluorescence spectroscopy for bound CTB. PBS without nanoparticles served as a background control (n = 3; mean ± SD). (B) DiD-labeled GM1-NPs and PEG-NPs were incubated with FITC-CTB, and imaged by fluorescence microscopy. (C) Increasing concentrations of FITC-CTB were incubated with the indicated fixed amounts of GM1-NPs, and specific binding was analyzed by fluorescence spectroscopy (n = 3; mean ± SD). (D) Increasing amounts of GM1-NPs were incubated with a fixed amount (10 μg/mL) of CTB, and specific binding was analyzed by fluorescence spectroscopy (n = 3; mean ± SD).

To determine the ability of GM1-NPs to bind CT, we incubated them, as well as control PEG-NPs, with fluorescently (FITC)-labeled CTB. Unbound CTB was removed by centrifugal filtration, and bound FITC was assayed by fluorescence spectroscopy. Over 95% of input CTB was bound to GM1-NPs, whereas the control PEG-NPs had only background levels of fluorescence (Fig 2A), showing the specificity of CTB binding to the GM1-NPs. We also constructed GM1-NPs and PEG-NPs in which a far-red fluorescent dye, DiD, was encapsulated in the PLGA core, and incubated them with FITC-CTB under the same conditions. Fluorescence imaging revealed co-localization of DiD and FITC in the GM1-NPs, whereas no FITC staining was observed in the PEG-NPs (Fig 2B). These results confirm that CTB binds specifically to GM1-NPs.

Toxin binding was concentration-dependent in regard to CTB and GM1-NPs (Fig 2C and 2D). Fitting of a one-site specific binding model revealed a maximal binding capacity (Bmax) of ~10^−7^ mol CTB per mg GM1-NP (equivalent to ~1 mg CTB/mg NP). Taken together, these results show that GM1-NPs are stable in a physiologically relevant salt solution, and can bind CTB in a specific and a high-capacity manner.

### Functional neutralization of cholera toxin by GM1 nanoparticle decoys

**Fig 3:**
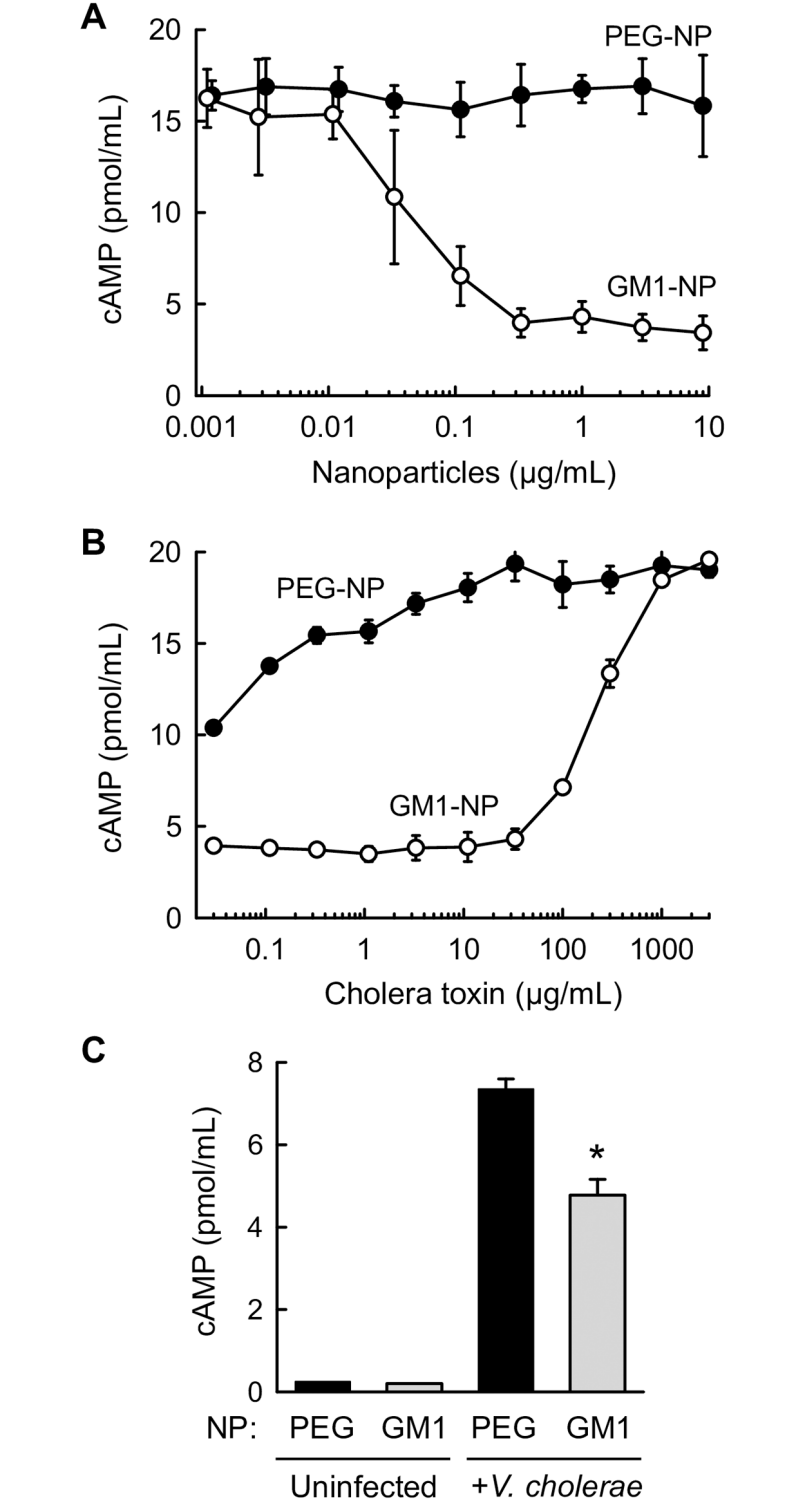
Neutralization of CT activity with GM1-NPs. (A) A fixed concentration (10 ng/mL) of CT was combined with increasing amounts of the indicated nanoparticles, and the mixtures were added to confluent monolayers of human HCA7 intestinal epithelial cells. After 2 h, levels of secreted cAMP were determined in the supernatants by ELISA (n = 3; mean ± SD). (B) A fixed amount (1 μg/mL) of the indicated nanoparticles were combined with increasing concentrations of CT, the mixtures were added to HCA7 monolayers for 2 h, and levels of secreted cAMP levels were measured by ELISA (n = 3; mean ± SD). (C) GM1-NPs or control PEG-NPs were added to HCA7 monolayers, which were then infected for 2 h with live *V*. *cholerae* or left uninfected, and secreted cAMP was determined (n = 3; mean ± SD; *p<0.05 vs PEG-NPs).

Having shown specific CTB binding to GM1-NPs, we next investigated whether the particles could block the functional impact of CT holotoxin on intestinal epithelial cells. A fixed concentration of CT was mixed with different concentrations of GM1-NPs or PEG-NPs, and the mixtures were added to monolayers of human HCA7 intestinal epithelial cells. As a functional read-out for CT bioactivity, we determined levels of secreted cAMP in the supernatants, which correlate closely with intracellular cAMP levels [25]. GM1-NPs neutralized the ability of CT to activate cAMP production and secretion in a concentration-dependent fashion, while the GM1-free control PEG-NPs had no effect (Fig 3A). Half-maximal neutralization of 10 ng/mL CT was achieved at 28 ng/mL GM1-NPs (as measured by their PLGA content). As a further demonstration of the specificity and saturability of the CT/GM1-NP interaction, we observed that increased CT concentrations could overwhelm the neutralizing capacity of GM1-NPs, so a CT concentration of 361 ng/mL was required in the presence of 1,000 ng/mL of GM1-NPs to recover 50% of the maximal CT response seen in the absence of nanoparticles (Fig 3B). Control PEG-NPs had no neutralizing effect under these conditions (although decreasing CT concentrations led to the expected diminishment of the epithelial cAMP response).

Neutralization of purified CT was a necessary precondition for practical utility of GM1-NPs, but the particles must be effective against CT produced by live bacteria to have therapeutic potential. Therefore, we infected HCA7 epithelial monolayers with live, CT-secreting *V*. *cholera*e in the absence or presence of nanoparticles, and measured cAMP secretion in the culture supernatants. GM1-NPs significantly attenuated the cAMP response compared to PEG-NPs, although attenuation was incomplete (Fig 3C). Nanoparticles alone without bacteria had no effect on cAMP production. Given the intense exposure of the epithelial monolayers to high numbers of bacteria without physiological mixing that occur with normal intestinal motility and the absence of a normal mucus layer, these data strongly suggest that the GM1-NPs can significantly neutralize CT produced by live bacteria in close contact with epithelial cells.

### Stability of GM1 nanoparticle decoys under physiologically relevant conditions

**Fig 4:**
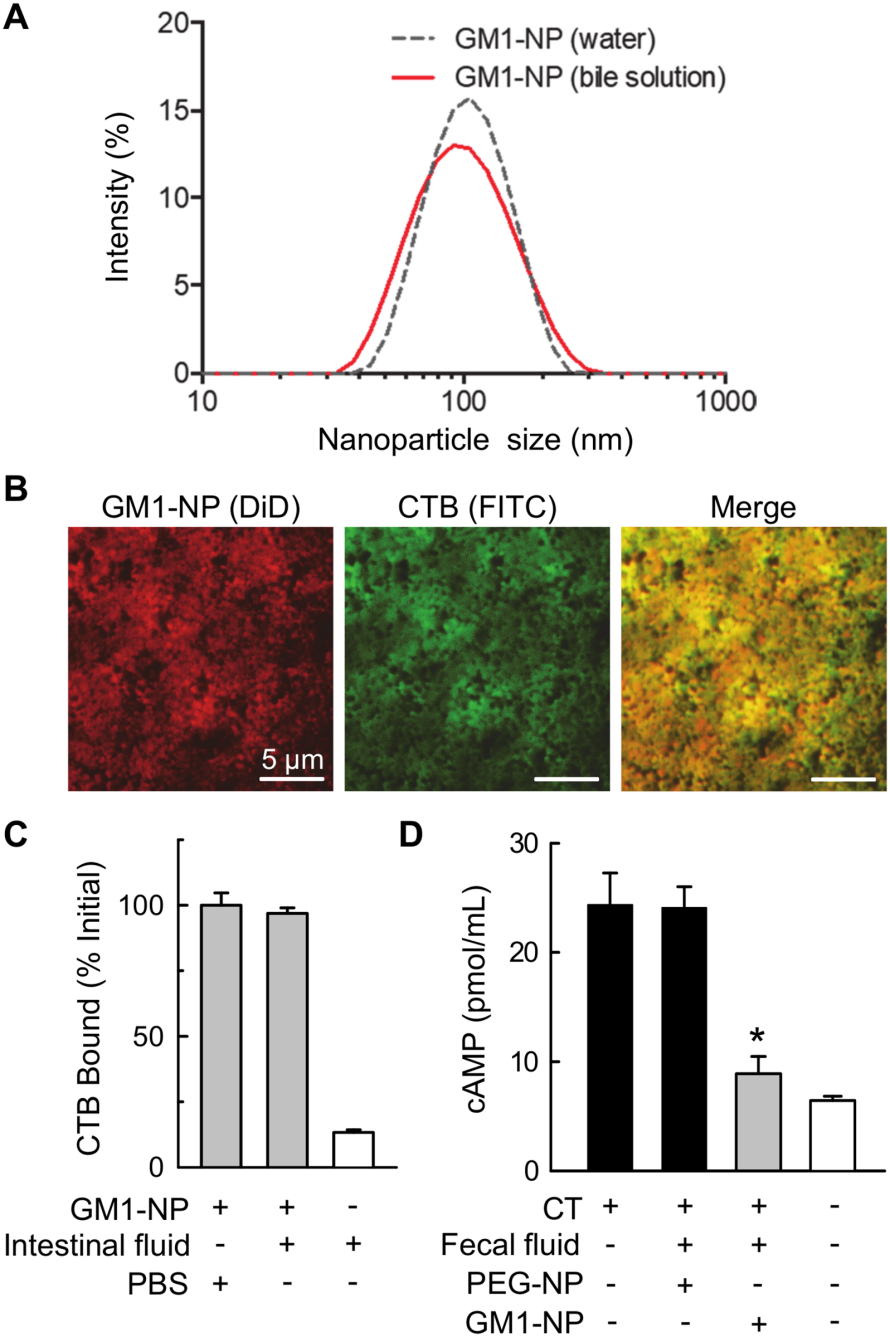
Functional stability of GM1-NPs in the presence of intestinal luminal factors. (A) Intensity-weighted size distribution of GM1-NPs incubated 30 min in distilled water or diluted porcine bile solution (1:16 dilution in water). (B) Fluorescence imaging of DiD-labeled GM1-NPs absorbed with FITC-CTB and incubated for 30 min in 1:16 diluted porcine bile solution. (C) GM1-NPs were loaded with FITC-CTB for 30 min, washed, and resuspended in luminal fluid from the small intestine of normal adult mice, or PBS as a control. After incubation for 24 h at 37°C, particle-bound and free FITC-CTB were separated by dialysis, and bound FITC-CTB was measured by fluorescence spectroscopy and related to the initial amount bound (mean ± SD, n = 3). Background readings were obtained with free FITC-CTB without GM1-NPs. (D) Fecal homogenates from mice were mixed 1:1 with GM1-NPs or PEG-NPs in culture media, and incubated for 1 h at 37°C, after which CT (10 ng/mL) was added for an additional 1 h before addition to HCA7 monolayers. After 2 h, cAMP levels in the supernatants were determined by ELISA (mean ± SD, n = 3; *p<0.05 vs PEG-NPs).

To be effective in the intestinal lumen, where *V*. *cholerae* resides and secretes CT, nanoparticles have to be stable and functional in the presence of the relevant physiological factors at that site. Of particular importance are bile acids whose amphoteric nature promotes lipid solubilization and digestive enzymes that can break down lipids and other complex molecules. Therefore, we tested whether GM1-NPs remained intact and active upon exposure to these luminal factors. Incubation of GM1-NPs in a solution containing concentrated porcine bile had no impact on particle size or their ability to bind FITC-labeled CTB (Fig 4A and 4B). Furthermore, incubation of CTB-loaded GM1-NPs for 24 h with luminal fluid (containing bile acids, various digestive enzymes, and some commensal bacteria) from the small intestine of mice did not detach the toxin, indicating that toxin binding to the nanoparticles was stable and not affected by luminal factors (Fig 4C; similar observations were made after 48 h of incubation). This conclusion was confirmed by the observation that exposure of the nanoparticles to fecal homogenates (which also contain bile acids and digestive enzymes, as well as large numbers of commensal bacteria and their enzymatic products) did not compromise the ability of GM1-NPs to functionally neutralize CT in respect to epithelial cAMP induction (Fig 4D). Together, these results demonstrate that the GM1-NPs display stable and prolonged functionality in the presence of intestinal luminal factors, suggesting that they are suitable for *in vivo* applications to neutralize CT.

### Attenuation of intestinal secretory response to CT and live *V*. *cholerae* by GM1 nanoparticle decoys *in vivo*

**Fig 5:**
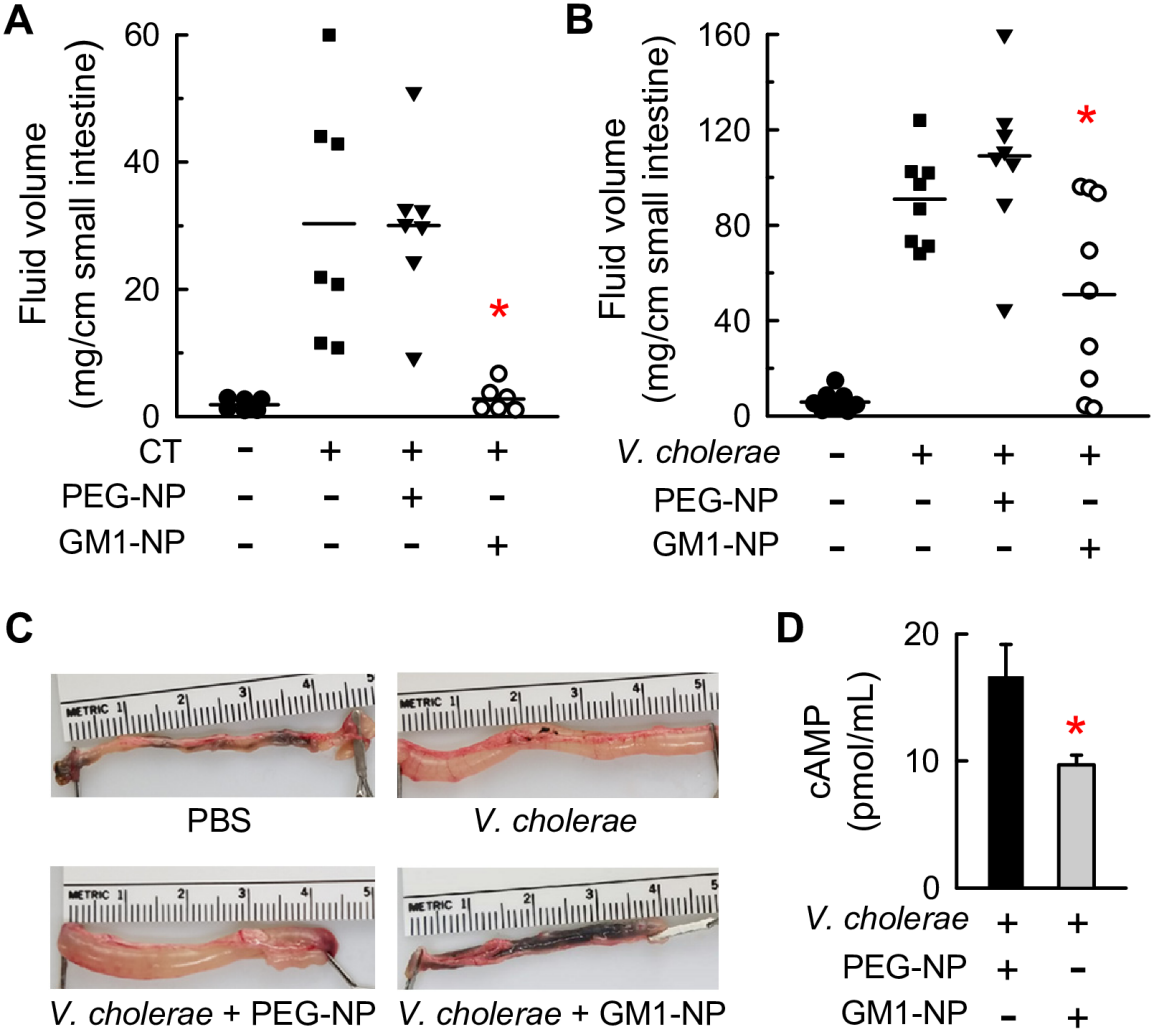
*In vivo* efficacy of GM1-NPs against CT and live *V*. *cholerae*. (A) Ligated intestinal loops were prepared in the distal small intestine of adult C57BL/6 mice, and injected with PBS as a control, or with 2.5 μg CT, without and with prior addition of GM1-NPs or control PEG-NPs. Fluid accumulation in the loops was determined after 4 h, and related to loop length (each point represents one animal, horizontal lines are geometric means; *p<0.05 vs PEG-NPs). (B) Loops were injected with PBS as a control, or live *V*. *cholerae* with GM1-NPs or control PEG-NPs. Fluid accumulation was determined after 16 h (each point represents one animal, horizontal lines are geometric means; *p<0.05 vs PEG-NPs). (C) Images of representative intestinal loops. (D) cAMP was measured in the luminal fluid collected from loops after injection of live *V*. *cholera*e with GM1-NPs or control PEG-NPs (n = 3; mean ± SD; *p<0.05 vs PEG-NPs).

To evaluate the therapeutic efficacy of the nanoparticles *in vivo*, we utilized ligated intestinal loops in adult mice as a model. Constructed in the distal small intestine, these loops allow undisturbed exposure of the intestine to defined microbial stimuli and therapeutic interventions in the physiologically relevant environment without confounding variables related to intestinal motility or variable susceptibility of adult mice to sustained infection with the target microbe. In a first test, we injected the loops with CT in the absence or presence of GM1-NPs or PEG-NPs. CT alone induced a robust fluid response in the lumen of the loops, which was not affected by the control PEG-NPs (Fig 5A). In contrast, GM1-NPs completely blocked the fluid response to CT, indicating that the nanoparticles were as effective *in vivo* as they were *in vitro*. Subsequently, we infected the loops with live *V*. *cholerae* with and without nanoparticles. Increased fluid secretion was observed after infection, which was significantly attenuated by treatment with GM1-NPs but not with control PEG-NPs (Fig 5B and 5C). In parallel to the attenuated fluid response, levels of secreted cAMP in the intestinal lumen were significantly decreased with GM1-NP treatment compared to control PEG-NPs after *V*. *cholerae* infection (Fig 5D). Neither of the nanoparticles had an impact on baseline fluid secretion without infection.

## Discussion

Cholera continues to be a major public health challenge in many regions of the world [5]. Medical strategies to combat this scourge can be divided into preventive approaches, which seek to protect individuals from infection, and therapeutic approaches, which attenuate disease symptoms in infected persons. For prevention, the FDA recently approved the first cholera vaccine, Vaxchora, composed of attenuated live bacteria, but the vaccine is currently only effective for *V*. *cholerae* serogroup 01 and, as a live agent, has the potential to cause disease itself, either in attenuated form in predisposed individuals or potentially upon reversion to a more virulent form [26]. In this regard, quality controls of live microbial agents as therapeutic agents can be challenging. As an alternative, attenuating medical strategies employ well-controlled interventions to ameliorate symptoms and assure survival while allowing mucosal immune defenses to clear the infection. The classical treatment is oral or intravenous rehydration [4], in which bacterially-induced diarrheal processes proceed unhindered, but the devastating systemic consequences of dehydration are prevented by providing sufficient electrolytes and water during the acute disease stage. Although usually effective for promoting survival, severe disease symptoms can still occur for days.

**Fig 6:**
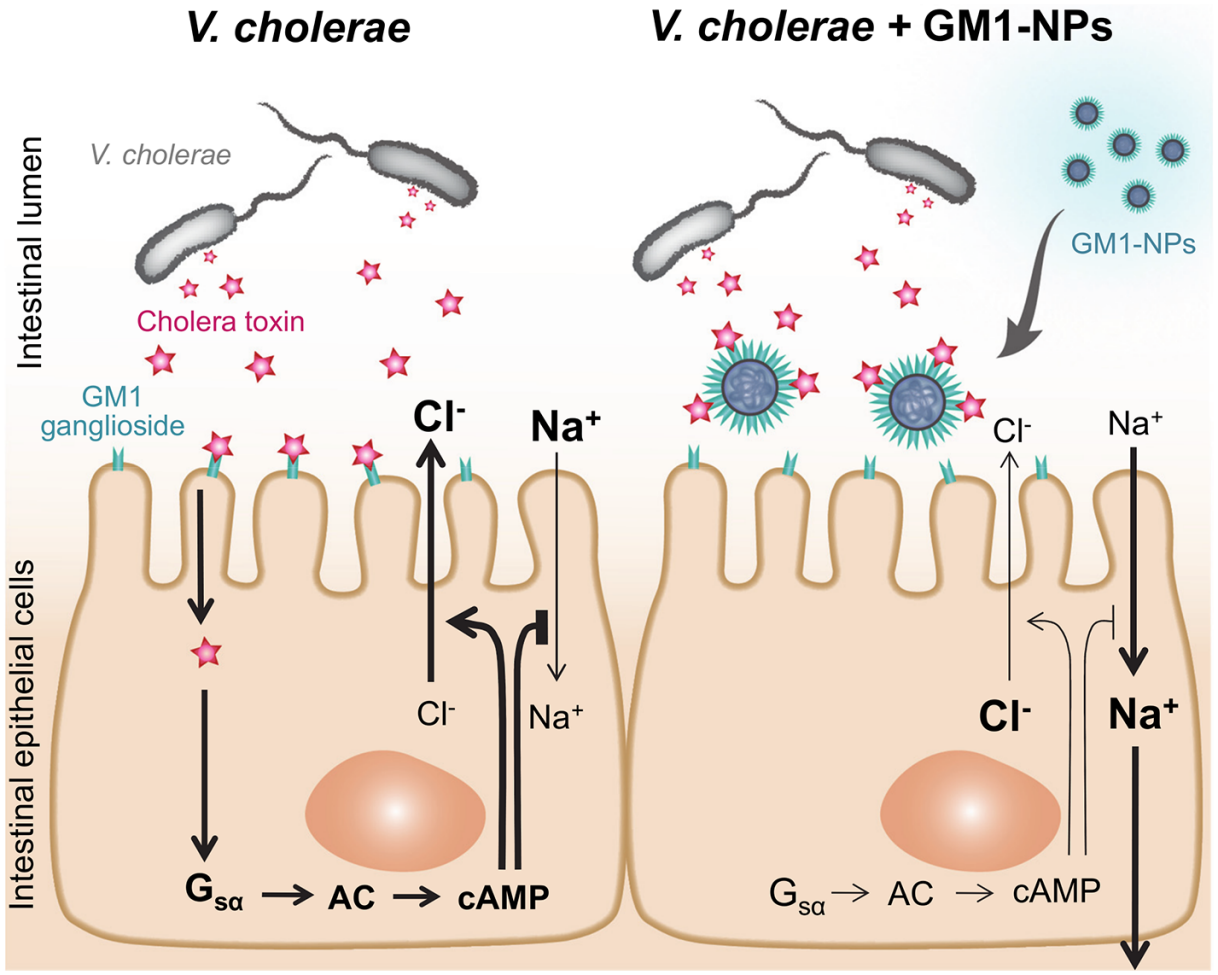
Model of therapeutic effect of GM1-NPs. GM1-coated nanoparticles act as decoys to absorb CT produced by *V*. *cholerae* before it can bind to epithelial cells to stimulate cAMP production and epithelial chloride secretion, and inhibit sodium absorption.

As an alternative attenuation strategy, we show here that a nanotechnology-based intervention can be effective at targeting the bacterially-produced CT, which is the primary cause of diarrheal symptoms in cholera [5]. By coating nanoparticles with the CT-binding lipid, GM1, the particles were able to bind and neutralize the toxin, thereby preventing its effects on epithelial electrolyte and fluid secretion *in vitro* and *in vivo* (Fig 6). This strategy represents a novel interventional approach whose mechanisms of action are physiologically distinct from vaccination, rehydration, or antibiotics, thus significantly broadening the medical armamentarium against cholera.

Neutralization strategies for microbial toxins have been proposed and implemented with different technological means, including antibodies and live bacteria. For cholera, *Escherichia coli* has been engineered to produce GM1 lipid on its surface [27]. The recombinant *E*. *coli* was shown to bind CT and attenuate *V*. *cholerae*-induced disease in animal models [15,27], thus underlining our findings. However, live microbes as therapeutic agents are problematic due to the difficulties in performing quality controls in manufacturing [28]. Furthermore, genetically engineered live bacteria, particularly those that belong to the normal intestinal microbiota, may colonize the intestine permanently [29], raising concerns about long-term microecological consequences of such interventions. In contrast, our nanoparticles were constructed exclusively from tractable reagents that can be readily quality-controlled. The fabrication process is effective and high-yield, thereby minimizing potentially offensive by-products and the accompanying need for extensive purification schemes. Furthermore, the nanoparticles do not replicate and are thus not retained for extended periods in the intestine beyond the treatment period.

In the nanoparticle design, we considered that many glycosylated lipid derivatives, such as gangliosides, contain sialic acid residues on their sugar chains that are prominently positioned and critical for attachment of cholera toxin [30]. Consequently, it was important that the GM1-oligosaccharides were located on the outside of the nanoparticle core in the proper orientation, so the lipid arrangement would resemble as much as possible the naturally occurring cell-surface location and affinity of GM1 to which CT normally binds. We were able to achieve the desired lipid location and orientation on the polymeric nanoparticle surface through a modified nanoprecipitation method [18,21], in which GM1-gangliosides with their inherent amphiphilic property self-assembled in a single-step synthesis on the hydrophobic nanoparticle surface to produce a lipid monolayer on the interface of the nanoparticle core and the hydrophilic GM1-oligosaccharides present on the outer shell.

The fabricated GM1-nanoparticles represent a novel class of core-shell structured lipid-polymer hybrid nanoparticles, which are known for combining the strengths of both liposomes and polymeric nanoparticles [18]. The lipid shell incasing the core mimics biological membranes and can mediate specific interactions with the environment, such as the reported interaction between the shell GM1 and soluble CT. Meanwhile, the nanoparticle core serves as structural support that provides controlled morphology, size tunability, and narrow size distribution [31]. In addition, lipid-polymeric nanoparticles have excellent physical stability [21,32,33], making GM1-NPs promising for their use in tropical environments. In terms of safety, PLGA polymer, which makes up the GM1-NP core, is a safe and FDA-approved biodegradable polymer [34], and GM1-ganglioside is extracted from natural cell membranes. Hence, it is likely that the GM1-nanoparticles are biocompatible and safe for prospective clinical translation.

Since cholera is often a disease affecting poor people in developing countries, cost-effective manufacturing is critical for clinical utility. PLGA has long been produced for pharmaceutical applications [35,36], and a variety of manufacturing processes have been used in industry for nanoparticle formulations [37–39]. Such processes could be adapted to large-scale GM1-NP production, facilitating downstream translational development. Optimal formulation will require further development, but the platform technology has great flexibility. For example, nanoparticles can be administered directly, as shown for formulations such as poly (lactic-co-glycolic acid) nanoparticles and polyacrylic acid nanoparticles to treat colitis or hypercalcemia [40,41]. Alternatively, nanoparticles could be loaded into capsules [42,43] or a pH-responsive polymer matrix for targeting to specific sections of the intestinal tract [44].

Our nanoparticle strategy has implications for treating other enteric infections in which microbially-produced toxins play an important and central pathophysiological role. For example, the heat-labile enterotoxin of enterotoxigenic *E*. *coli*, the most common cause of traveler’s diarrhea, also binds GM1 ganglioside [45]. Structural analysis of CTB and the binding subunit of LT bound to GM1-pentasaccharide revealed that the residues contacting the terminal galactose sugar are conserved between the two toxins [46], suggesting that nanoparticle-based intervention would predictably also be effective in enterotoxigenic *E*. *coli*-induced disease. For other toxin-mediated enteric diseases, such as those caused by shiga toxin from *Shigella dysenteriae* [47], similar nanotechnology strategies using the appropriate lipids may also be a promising new therapeutic avenue.
